# Inflammatory Bowel Disease: Genetics, Epigenetics, and Pathogenesis

**DOI:** 10.3389/fimmu.2015.00551

**Published:** 2015-11-02

**Authors:** Italia Loddo, Claudio Romano

**Affiliations:** ^1^Inflammatory Bowel Disease Unit, Pediatric Department, University of Messina, Messina, Italy

**Keywords:** inflammatory bowel disease, genetics, epigenetics, IBD, Crohn’s disease, early-onset IBD

## Abstract

Inflammatory bowel diseases (IBDs) are complex, multifactorial disorders characterized by chronic relapsing intestinal inflammation. Although etiology remains largely unknown, recent research has suggested that genetic factors, environment, microbiota, and immune response are involved in the pathogenesis. Epidemiological evidence for a genetic contribution is defined: 15% of patients with Crohn’s Disease (CD) have an affected family member with IBD, and twin studies for CD have shown 50% concordance in monozygotic twins compared to <10% in dizygotics. The most recent and largest genetic association studies, which employed genome-wide association data for over 75,000 patients and controls, identified 163 susceptibility loci for IBD. More recently, a trans-ethnic analysis, including over 20,000 individuals, identified an additional 38 new IBD loci. Although most cases are correlated with polygenic contribution toward genetic susceptibility, there is a spectrum of rare genetic disorders that can contribute to early-onset IBD (before 5 years) or very early onset IBD (before 2 years). Genetic variants that cause these disorders have a wide effect on gene function. These variants are so rare in allele frequency that the genetic signals are not detected in genome-wide association studies of patients with IBD. With recent advances in sequencing techniques, ~50 genetic disorders have been identified and associated with IBD-like immunopathology. Monogenic defects have been found to alter intestinal immune homeostasis through many mechanisms. Candidate gene resequencing should be carried out in early-onset patients in clinical practice. The evidence that genetic factors contribute in small part to disease pathogenesis confirms the important role of microbial and environmental factors. Epigenetic factors can mediate interactions between environment and genome. Epigenetic mechanisms could affect development and progression of IBD. Epigenomics is an emerging field, and future studies could provide new insight into the pathogenesis of IBD.

## Introduction

Inflammatory Bowel Diseases (IBDs) are complex, multifactorial disorders characterized by chronic relapsing intestinal inflammation. The two major subtypes of IBD are Ulcerative Colitis (UC) and Crohn’s Disease (CD).

Ulcerative Colitis and CD are important worldwide health problems, with an incidence in Europe of 12.7 and 24.3 per 100,000 person-years, respectively, and prevalence of 0.5 and 1.0%.

The incidence of IBDs is continually growing among children and adults, all over the world (1).

**Figure 1:**
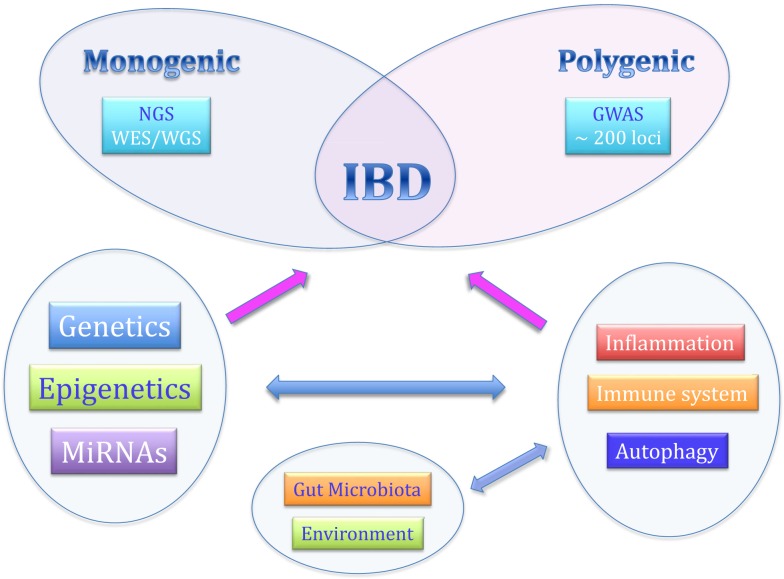
**The complex pathogenesis of inflammatory bowel diseases**.

Although the exact etiology is still not completely known, recent studies have indicated that personal genetic susceptibility, environment, intestinal microbiota, and immune system are all involved in the pathogenesis of IBDs (Figure 1).

The heritability model of CD and UC is still unknown, but many genetic and environmental factors are likely to be involved. These conditions tend to occur in multiple familial cases, and having an affected relative is an important risk factor for the onset of IBD.

Population-based studies have provided compelling evidence that genetic factors contribute to the pathogenesis of IBD; an 8- to 10-fold greater risk of IBD among relatives of UC and CD probands has been demonstrated and, most importantly, that there is concordance between twins (2).

Twin studies have provided the best evidence for genetic predisposition to IBD, which is stronger for CD than UC. In particular, twin and family studies for IBD have shown that a child has a 26-fold increased risk for developing CD when another sibling already has it, and the risk is ninefold increased in the case of UC (3).

However, the fact that genetic factors account for only a part of general disease variance indicates that microbiota and environment may interact with genetic susceptibility in the pathogenesis of IBD.

The adaptive immune system has been classically considered to play the main role in the pathogenesis of IBD. Recent research in genetics and immunology has confirmed that the innate immune system maintains great importance in inducing gut inflammation.

New advances in understanding IBD pathogenesis explain important disease mechanisms, including not only the innate and adaptive immunity, but also interactions between genetic, microbial, and environmental factors (4).

## Genetics and Pathogenesis

Over the past few decades, there have been important advances in our understanding of genetic contributions to IBD. This is due to technological progress in genetic testing and DNA sequencing that has allowed many genome-wide association studies (GWAS), which have identified new single nucleotide polymorphisms (SNPs) (4, 5).

Nucleotide-binding oligomerization domain containing 2 (NOD*2*) was the first susceptibility gene for CD discovered in 2001. This gene codes for a protein that acts as an intracellular receptor for bacterial products in monocytes and transduces signals leading to NFkB activation. The activation of NOD2 with muramyl dipeptide induces autophagy in dendritic cells (DCs). DCs from CD patients with susceptibility variants in NOD2 gene are deficient in autophagy induction and also show reduced localization of bacteria in autophagolysosomes (6).

Genome-wide association studies have identified and confirmed many susceptibility loci for IBDs. The identification of susceptibility loci has enhanced understanding of causes of the disorder by providing important clues in crucial and disturbed pathways of the intestinal immune system (7).

Genetic analyses have reported two other autophagy-related genes, *IRGM* and *ATG16L1*, showing an important role for autophagy in immune responses in IBD. Genetic variants that have been found to confer an increased risk of CD indicate the importance of innate immunity, autophagy, and phagocytosis in its pathogenesis.

Other genes, like *IL23R* and *PTPN2*, are also associated with autoimmune disease, suggesting another aspect of Crohn’s pathogenesis.

Recent progress in the genetics of IBD can explain the underlying pathogenesis of the disease.

Conventional IBD is a group of polygenic disorders in which hundred(s) of susceptibility loci contribute to the overall risk of disease.

The most recent and largest genetic association study, which employed genome-wide association data for over 75,000 adolescent and adult-onset IBD patients and controls, identified 163 susceptibility loci for IBD, encompassing ~300 potential candidate genes. Of these 163 loci, 110 conferred risk to both IBD subtypes, whereas 30 loci where unique to CD and 23 loci were unique to UC. More recently, a trans-ethnic analysis including over 20,000 individuals of European and non-European ancestry identified an additional 38 new IBD loci, highlighting shared genetic risk across populations and increasing the number of known IBD risk loci to 200 (8, 9).

The evidence of shared IBD risk loci across diverse populations suggests that combining genotype data from cohorts of different ancestry will enable the detection of additional IBD-associated loci.

However, all identified loci individually contribute only a small percentage of the expected heritability in IBD (3).

The increasing number of susceptibility gene loci described in IBD indicates that genetic components are important factors involved in the disease pathogenesis. Identified genetic factors account for only a small proportion of the disease variance: 13.1% for CD and 8.2% for UC. Overall, explainable susceptibility loci and genetic risk factors discovered so far, account for only 20–25% of the heritability (genetic risk) (8, 10).

## Monogenic Forms and Very Early-Onset IBD

Approximately 20–25% of patients with IBD are diagnosed before 16 years old. The onset of intestinal inflammation in children can affect development and growth. Age of onset can also provide information on type of IBD and associated genetic features.

Although most cases of IBD are correlated with a polygenic contribution toward genetic susceptibility, there is a spectrum of rare genetic disorders that produce IBD-like intestinal inflammation (11).

Early-onset inflammatory bowel diseases (EO-IBD) (before 5 years) and very-early-onset inflammatory bowel diseases (VEO-IBD) (before 2 years) are rare, particularly severe disease presentations. Few case series have reported on early-onset of IBDs, often described as CD or CD-like, but starting within the first year of life with typical characteristics of CD. The majority of VEO-IBD are caused by genetic defects (monogenic diseases).

The genetic variants that cause these disorders have a wide effect on gene function. These variants are so rare in allele frequency that the genetic signals are not detected in GWAS of patients with IBD. With recent advances in genetic mapping and sequencing techniques and increasing awareness of the importance of these rare disorders, ~50 genetic disorders have been identified and associated with IBD-like immunopathology.

Monogenic defects have been found to alter intestinal immune homeostasis via several mechanisms.

These include disruption of the epithelial barrier and the epithelial response, as well as reduced clearance of bacteria by neutrophil granulocytes and other phagocytes. Other single-gene defects induce hyperinflammation or autoinflammation or disrupted T- and B-cell selection and activation.

Hyperactivation of the immune response can result from defects in immune inhibitory mechanisms, such as defects in IL-10 signaling or dysfunctional regulatory T-cell activity (3).

The main of the IL-10 pathway within the colonic mucosa is confirmed by the occurrence of severe colitis during the first weeks of life in infants carrying mutations in *IL10*, *IL10RA*, or *IL10RB* genes. Immunosuppression has failed to correct the defect in this pathway, which seems to be important to control colon inflammation (12).

Loss-of-function defects in IL-10 and its receptor (encoded by *IL10RA* and *IL10RB* genes) cause VEO-IBD with perianal disease and folliculitis within the first months of life. All patients with loss-of-function mutations that prevent IL-10 signaling develop IBD-like immunopathology, indicating that these defects are a monogenic form of IBD with 100% penetrance (13, 14).

A functional IL-10 pathway is essential for immune homeostasis within the colon, and defects in this cytokine or one of the subunits of its receptors cause extensive inflammation of the colon and perianal region. Thus, on a clinical basis, we can suspect a defect in the IL-10 pathway in children, presenting deep ulcerations and granuloma (CD-like), especially in consanguineous families (12).

It is a challenge to diagnose the rare patients with monogenic IBD, but accurate genetic diagnosis is important for assessing prognosis, and proper treatment of patients.

This group of diseases has high morbidity, and subgroups have high mortality if untreated. Based on the causes, some require different treatment strategies from most cases of IBD. A genetic diagnosis should always be carried out due to the differences in prognosis and medical management (3).

## IBD in the ERA of Next-Generation Sequencing

Next-generation sequencing (NGS) is a new technology with the potential to identify every genetic variation throughout the human genome in a single experiment.

Next-generation sequencing technologies have revolutionized the field of medical genetic research and are currently being used to search for Mendelian disease genes, and applied for the diagnosis of patients with genetically heterogeneous disorders. This can be performed much faster and more cost efficiently than with traditional techniques.

It is often a less expensive option than traditional Sanger sequencing for diseases characterized by genetic heterogeneity. This is a novel approach to discover lower prevalence with higher effect size (15).

Because of the large amount of data that are being generated, bioinformatic analysis plays an important role in research and diagnostics (16, 17).

The two sequencing approaches for detecting variations in the genetic code are whole genome sequencing (WGS) and whole exome sequencing (WES).

Whole genome sequencing is the ultimate approach for detecting all genomic variations in a patient’s genome. However, current NGS instruments are limited in terms of throughput and cost efficiency and this approach is often only used for large-scale research studies and gene discovery projects.

Whole exome sequencing is a more cost-efficient strategy for novel disease gene discovery and diagnostics in human genetics. The protein-coding regions constitute the exome, ~1% of the human genome or ~30 Mb, split across ~180,000 exons. Currently, the great majority of mutations responsible for Mendelian diseases in humans affects sequences within the coding regions of exons or are located within a few nucleotides of the exon boundaries. Exome sequencing basically refers to the enrichment of sequences corresponding to all (or nearly all) protein-coding exons followed by next-generation sequencing.

Exome sequencing was introduced in 2009. Since then, it has been used to discover several hundred novel disease genes and has begun to significantly improve diagnostics for patients with rare genetic diseases. It has rapidly become one of the main tools for studying the genetic causes of diseases and we have learned much from exome sequencing.

Whole exome sequencing covers only coding areas of the genome, and costs less than WGS, providing higher-depth coverage and therefore greater certainty regarding novel discoveries. But only together with subsequent functional studies on identified proteins and pathways will novel technologies elucidate underlying pathogenic mechanisms.

Use of WES in clinical diagnostics has grown significantly since clinical laboratories started performing it. Many patients with rare recessive and dominant disorders, who had previously spent years on an uninformative diagnostic odyssey, have now had diagnoses made through WES (16).

Interestingly, results of diagnostic applications of NGS indicate that there is a much wider phenotypic spectrum associated with mutations in many genes than was suspected from initial clinical definition and Sanger sequencing.

The advent of NGS techniques has allowed new large-scale approaches with unexpected diagnostic power. In particular, WES is changing the diagnostic paradigm in medical genetics practices.

The traditional diagnostic approach with clinical evaluation and laboratory analysis provides a diagnosis in ~50% of patients. WES offers the possibility to have an answer in most of the remaining cases, providing diagnosis in another 25–30% of patients (18).

The WES approach has been successfully used to identify single variants in very early-onset IBD and has been quite successful in elucidating novel monogenic forms of IBD and new susceptibility genes. However, many polymorphisms that affect disease susceptibility are located in non-coding areas of the genome: for this reason, the ENCODE (Encyclopedia of DNA Elements) project has highlighted the importance of non-coding regions in disease risk, trying to identify all functional elements in the human genome sequence (19).

## Epigenetics and IBD

Inflammatory bowel diseases could be caused by interactions between the patient and the environment, in particular the genome, the immune system, the intestinal microbiota, and specific environmental factors such as the effects of breastfeeding, food, smoking, drugs, and so on. Epigenetics may be defined as mitotically heritable changes in gene expression without altering the DNA sequence.

Gene expression can be altered by changes to the structure and function of chromatin. Different cells in the body are characterized by different functions and different levels of gene expression despite each sharing the same genetic code. This variation in gene activity from cell to cell is achieved by mechanisms and processes that are collectively termed epigenetics. The main epigenetic mechanisms include DNA methylation, histone modification, RNA interference, and the positioning of nucleosomes. These epigenetic mechanisms, in particular DNA methylation, appear to be very important in the interaction between environment and genome. It is potentially reversible and heritable over rounds of cell division (20). Variation in DNA methylation is a well-recognized cause of human disease and is likely to play a pivotal role in the cause of complex disorders. Several well-known disorders of imprinting are known including Beckwith–Wiedemann syndrome, Temple syndrome, Wang–Ogata syndrome, Silver–Russell syndrome, Angelman syndrome, and Prader–Willi syndrome. Imprinted genes are thought to play an important role in fetal growth and their carefully regulated expression is important for normal cellular metabolism and human behavior.

The challenge is to identify consistent epigenetic alterations of etiological significance, given that epigenetic modification of DNA differs between tissues, occurs at different times of development within the same tissue and is sensitive to continual environmental factors.

Epigenetics has developed into one of the most promising concepts in all areas of biomedical research. Recent epigenetic studies have shown that interactions between genome and environment play an important role in the phenotypical expression of diseases, explaining also the differences in disease expression in monozygotic twins (1).

Much evidence supports the idea that IBD is caused by a complex interaction between genetic mutations of multiple genes and environmental factors. There is growing evidence that epigenetic factors can play an important role in the pathogenesis of IBD.

A number of potential clinical applications of epigenetics in diagnostics and therapeutics are receiving attention. The diagnostic applications of epigenetics include the use of biomarkers to confirm diagnosis, stratify disease course and response to chemotherapy, and predict development of cancer (11).

DNA methylation is the most studied epigenetic modification and during the last decade its correlation to IBD pathogenesis has been well established. Several reports have suggested that there are significant differential DNA methylation statuses between normal and inflamed tissues from CD and UC patients. Therefore, there are evidences that the hypermethylation of many gene promoters is associated with IBD patients.

Genes from different molecular pathways have been studied but till now there is no standardized database of methylated genes in IBD.

DNA methylation patterns have proven to be most useful in the sensitive detection of disease.

DNA-methylation-based technologies have a promising future in both clinical diagnostics and therapeutics. DNA methylation markers have been developed using targeted candidate gene approaches and have applications in diagnostics, but can also contribute to therapeutics as predictors of therapeutic response (20). Further studies of epigenetic factors associated with IBD could lead to new therapeutic strategies, whether they specifically target epigenetic mechanisms or affect the pathways they control.

DNA methylation should be studied in depth to understand the molecular pathways of IBD pathogenesis, and epigenetic studies of IBD discussed that may have a significant impact on the field of IBD research.

## The Emerging Role of Mirna in IBD

The field of microRNA (miRNA) research is expanding rapidly. MiRNAs are strongly implicated in the pathogenesis of many common diseases, including IBDs, playing an important role in the development, regulation and differentiation of the innate and adaptive immune system (21).

MicroRNAs are a class of endogenous small non-coding single-stranded RNA molecules, ~18–24 nt long, encoded in genomic DNA, which act as post-transcriptional regulators of gene expression. The biogenesis of miRNAs goes from transcription in the nucleus to generation of the mature miRNA in the cytoplasm.

MiRNA genes are located throughout the genome, either within intronic sequences of protein-coding genes, within intronic or exonic regions of non-coding RNAs, or set between independent transcription units (intergenic). Some miRNAs have their own promoters and are transcribed independently, some share promoters with host genes, while others are co-transcribed as a single primary miRNA transcript (21, 22).

It is estimated that miRNAs regulate more than 60% of protein-coding mRNAs and that more than one-third of human genes are targets for miRNA regulation.

In particular, each miRNA can target hundreds of mRNAs and each mRNA can be regulated by several miRNAs, resulting in mRNA destabilization and/or inhibition of translation.

They regulate important cellular functions such as differentiation, proliferation, signal transduction, and apoptosis and exhibit highly specific regulated patterns of gene expression.

Emerging evidence suggests the regulation of miRNAs expression through epigenetic mechanisms such as DNA methylation, histone modifications, and circular RNAs (circRNAs). DNA methylation, the addition of methyl groups at CpG islands by DNA methyltransferases, is associated with transcriptional repression. Similarly, acetylation or deacetylation of histones may alter transcriptional activity. This process adds complexity to our understanding about regulation of gene expression (21).

Although a large number of miRNAs have been identified, little is known about their function (23).

There is growing evidence that miRNAs play a role in the induction of cancer, inflammatory, and autoimmune diseases. In the intestinal tract, miRNAs are involved in tissue homeostasis, intestinal cell differentiation, and the maintenance of intestinal barrier function (24).

Nowadays, research is interested in the possibility to use miRNAs as biomarkers and therapeutic target in IBD. There are emerging data from human diseases studying miRNAs as novel biomarkers in diagnosing and predicting disease course and response to therapy.

Recently, several studies have shown a differential expression of miRNAs in tissue samples and blood from patients with IBDs compared with healthy controls, suggesting that miRNAs may be considered as novel biomarkers of these diseases (25).

Given that CD and UC differ in their clinical presentations, genetic associations, gene expression patterns, and immune responses, differing miRNA profiles are expected for these two conditions.

Studies to date have identified unique miRNA expression profile signatures in IBD and preliminary functional analyses associate these deregulated miRNAs to canonical pathways associated with IBD pathogenesis (26).

Crohn’s Disease and Ulcerative Colitis patients have unique miRNA expression profiles in their target organs. While some differentially expressed miRNAs are common to other immune-related disorders, most are unique.

Moreover, it has been shown that CD and UC differ not only in their tissue miRNA profiles, but also in their peripheral blood miRNA profiles. Then there are unique miRNA tissue profiles and distinct miRNA profiles in peripheral blood.

While UC and CD represent distinct diseases with some overlap, identification of distinct miRNA expression profiles may provide an early method to determine a patient’s disease course. After the functional consequences of alterations in miRNA expression are established, miRNA may also become the target of future treatments.

Further investigation about the roles of miRNAs in the human context will improve our knowledge of miRNAs in the pathogenesis and diagnosis of IBD and will be useful for the development of miRNA-based therapies (27).

## Conclusion

The number of potential IBD susceptibility genes continues to increase. The increasing number of genetic loci associated with IBD requires other studies to understand how they involve immunity and inflammation in susceptible individuals.

The identification of genetic variants may define a specific disease phenotype to help follow clinical progression and eventually develop new targeted therapies.

However, the evidence that genetic factors contribute in small part to disease pathogenesis confirms the important role of microbial and environmental factors.

Epigenetic mechanisms may affect development and progression of IBD, mediating interactions between genome and environment. Epigenomics is an emerging field, and future studies could provide new insight into the pathogenesis of IBD.

The role of miRNA in IBD represents a new pathway for discovery of disease mechanisms, diagnostics, and therapeutics.

These new discoveries on the genetics of IBD imply that future research on interactions between genes and between genes and environment will be essential to better understand the pathogenesis of these diseases and more appropriate medical therapy.

## Conflict of Interest Statement

The authors declare that the research was conducted in the absence of any commercial or financial relationships that could be construed as a potential conflict of interest.
